# Commentary: Developing research knowledge and capability in undergraduate nurses: evaluation of targeted placements

**DOI:** 10.1177/17449871211004357

**Published:** 2021-07-21

**Authors:** Natalie Elliott

**Affiliations:** Year 3 Nursing Student (Adult field), Glasgow Caledonian University, UK

This paper evidences that experiential learning within clinical research is beneficial for the development of a research interested and active workforce. It also demonstrates that this process can have a positive impact on the quality of practice in the organisations. Having spent one of my student nurse placements within a clinical research facility, along with having an appreciation of research and the value it brings, I was interested to read the results of this research.

It is well known that many student nurses dislike the evidence-based practice theory modules as they are unable to relate the underpinning theory to practice (Ferguson et al., 2017). Additionally, within Jeppesen et al.’s (2017) systematic review, it is noted that students are motivated by practical teaching where simulated learning enables them to easily integrate the taught theory to clinical practice. However, the importance of research in nursing is a key component of the Nursing and Midwifery Code (Nursing and Midwifery Council, 2018) in the UK as research enables nurses to bring safe, innovative and effective practice to patients. Additionally, it is a nurse’s professional obligation to be able to critically assess the evidence that they use to underpin their practice. Therefore, it is imperative that pre-registration students’ perceptions of research are changed, and they feel confident about its significance and empowered to use it.

In the case of this paper, the results found that 100% of the students, who completed the questionnaire, enjoyed the research placement, with 65% of the students stating they would consider post-graduate study because of this experience. This is noteworthy, as it would appear self-evident that as the number of nurses involved in research increases, the knowledge base of the profession is enhanced, along with creating an inquisitive culture which does not accept the status quo and actively strives for improvement. Interestingly, the study also found that a clinical research placement encouraged students to scrutinise practice, especially when the organisation supports open dialogue about improving the culture, ultimately leading to an improvement in patient safety and quality of patient care.

Research confident professionals are integral to the nursing profession and the foundations should commence with pre-registration students (Council of Deans of Health, 2019). However, I am left wondering if the way research modules are taught to nursing students is relatable enough to cultivate their confidence. Perhaps more participatory and practical learning methods, such as involvement in clinical research placements or exposure to secondary research projects (not merely literature reviews), would foster a positive influence on students’ perceptions and engagement with research? I believe there would be advantages to replicating this study elsewhere to assess the generalisability of the results.

Overall, this paper contributes in expanding the literature on a clinical research placement for pre-registration nursing students, whilst also providing a valuable depiction of the benefits that it provides to both the individual student, the nursing profession and the wider healthcare organisation and its stakeholders.
